# A Novel Dual-Band (38/60 GHz) Patch Antenna for 5G Mobile Handsets

**DOI:** 10.3390/s20092541

**Published:** 2020-04-29

**Authors:** Marwa H. Sharaf, Amira I. Zaki, Radwa K. Hamad, Mohamed M. M. Omar

**Affiliations:** Electronics and Communications Department, College of Engineering and Technology, Arab Academy for Science, Technology & Maritime Transport, Alexandria 21937, Egypt; amzak10@aast.edu (A.I.Z.); radwa_hamad@aast.edu (R.K.H.); abuahmad.omar@aast.edu (M.M.M.O.)

**Keywords:** MIMO, 5G mobile handsets, dual-band antenna, microstrip patch antenna, millimeter-wave

## Abstract

A compact dual-frequency (38/60 GHz) microstrip patch antenna with novel design is proposed for 5G mobile handsets to combine complicated radiation mechanisms for dual-band operation. The proposed antenna is composed of two electromagnetically coupled patches. The first patch is directly fed by a microstrip line and is mainly responsible for radiation in the lower band (38 GHz). The second patch is fed through both capacitive and inductive coupling to the first patch and is mainly responsible for radiation in the upper frequency band (60 GHz). Numerical and experimental results show good performance regarding return loss, bandwidth, radiation patterns, radiation efficiency, and gain. The impedance matching bandwidths achieved in the 38 GHz and 60 GHz bands are about 2 GHz and 3.2 GHz, respectively. The minimum value of the return loss is −42 dB for the 38 GHz band and −47 for the 60 GHz band. Radiation patterns are omnidirectional with a balloon-like shape for both bands, which makes the proposed single antenna an excellent candidate for a multiple-input multiple-output (MIMO) system constructed from a number of properly allocated elements for 5G mobile communications with excellent diversity schemes. Numerical comparisons show that the proposed antenna is superior to other published designs.

## 1. Introduction

Fifth Generation (5G) technology has been a matter of controversy due to the enormous increase of users connected with their smartphones to the network relative to the scarcity of the available current bandwidth of the Fourth Generation (4G) technology [1]. This has led to the severe need for larger capacity and faster data rates [2,3,4,5,6], which are currently 100 times faster, and are expected to be 1000 times faster by 2030 [7]. Wireless channel capacity can be increased without the necessity for additional spectra or power in environments rich in scattering when introducing 5G mobile communication techniques [8,9,10]. This can be applied by increasing the number of antennas at the transmitter and/or the receiver of the wireless link [11,12,13,14,15,16,17,18,19]. As a consequence of requiring multiple antennas in a compact system like a mobile handset, the single antenna should be compact and operate at different required bands as required [20,21]. The present paper offers a solution for such low profile antennas operating at 38 and 60 GHz frequency bands.

The unused millimeter-wave electromagnetic spectrum (30–300 GHz) has attracted attention due to its multi-gigabit/s transmission rate exploiting widely available bandwidth to meet the demands of 5G applications which require high quality and low latency transmission [22,23,24,25,26]. The frequency bands centered at 28, 38, 60, and 73 GHz have been allocated for 5G mobile communications by the International Telecommunications Union (ITU) [27]. Bands of 59–64 GHz are allocated by the Federal Communications Commission (FCC) as an unlicensed band for short-range and wireless communications at high speeds [28]. The frequency band around 60 GHz has particular importance because of the wide 7–9 GHz frequency range of unlicensed spectra available. However, significant attenuation is caused by oxygen molecules in the atmosphere to react with radio frequency (RF) signals, reaching up to 10 dB/km. Due to this defect, the 60 GHz band is not a suitable band for wireless communication applications and long-range radar. For this reason, the dual-band (38/60 GHz) microstrip patch antenna proposed in the present work could be a good solution for forthcoming 5G antenna systems that utilize the 60 GHz frequency band, and provides the 38 GHz frequency band (a potential candidate for next generation communication) as an additional operational band due to its low oxygen absorption rates relative to the 60 GHz band [29].

Recently, a significant amount of research work has provided numerous designs for mobile handset antennas for 5G applications. In [29], a 28 GHz four-port MIMO antenna is proposed, where each antenna has an end-fire gain of about 10 dBi by employing an array of metamaterial unit cells. The work of [30] introduces a 60 GHz antenna that consists of H-shaped and E-shaped slots on the radiating patch. A two-port 5G dual-band (28/38 GHz) MIMO antenna system is proposed in [31]. This antenna is realized using two arrays, each consisting of three elements. In [32], a dual-band (38/54 GHz) microstrip patch antenna is proposed. The work of [27] proposes a 38 GHz slotted patch antenna for 5G wireless applications. A circularly polarized magneto-electric dipole antenna with high efficiency based on printed ridge gap waveguide is proposed in [33] to operate in the frequency range of 29.5–37 GHz. The work of [34] presents a compact antenna design with polarization and pattern diversity operating in the frequency band (34–38 GHz) for MIMO-based 5G mobile communication systems.

The present work proposes a novel (38/60 GHz) dual-band microstrip patch antenna for 5G mobile phones. The proposed patch can be used either as a single antenna or an element to construct compact MIMO antenna systems. In addition, due to the very weak coupling between adjacent elements of the proposed dual-band antenna, it can be used to construct high-gain compact arrays for 5G applications requiring smart antennas with capabilities of beam steering and detection of direction of arrival. The proposed antenna is composed of two patches: the first is inset-fed by a microstrip line, whereas the second is fed using both capacitive and inductive coupling to the first patch. The dimensions of the first patch are set so that the patch operates at the 38 GHz band, whereas the second patch is designed to operate at the 60 GHz band. Combining these two bands in a single compact antenna will save the power and space needed in a mobile handset. The radiation patterns produced by this antenna are shown to be suitable for 5G mobile communications. It should be noted that the electromagnetic simulations presented in the present work were performed using the commercially available CST Studio Suite^®^ software package.

The novelty of this work lies in the duality usage of the 38 and 60 GHz bands. This is unprecedented in previous work.

The remaining part of the paper is organized as follows. Section 2 describes the evolutionary design (stages) and an explanation of the principles of operation of the dual-band microstrip patch antenna proposed for 5G mobile handsets. Section 3 displays and discusses the obtained numerical results and the experimental measurements concerning the performance assessment of the proposed patch. Some comparisons with the results of other published work are presented for comparative assessment. Finally, Section 4 is devoted to the main conclusions of this work. 

## 2. The Proposed Dual-Band Microstrip Patch Antenna

**Figure 1:**
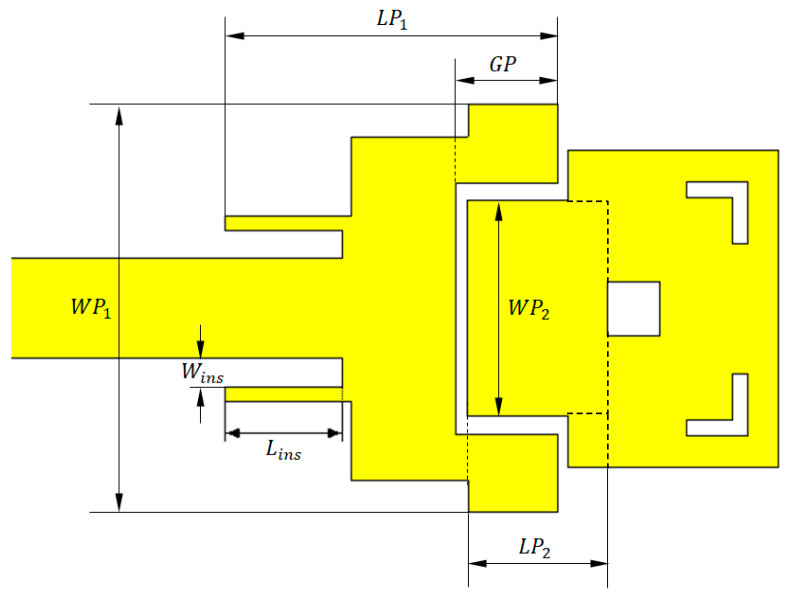
Geometry of the proposed dual-band (38/60 GHz) microstrip patch antenna.

The proposed dual-band microstrip patch antenna is shown in Figure 1. The proposed antenna is based on the design of two patches operating at different bands. The first patch is excited directly by a microstrip line through an inset feed, whereas the second patch is fed indirectly through a capacitive (edge) coupling with the first patch. The dimensions of the first patch are set so that the patch operates at 38 GHz, whereas the dimensions of the second patch are designed to operate at 60 GHz.

At 38 GHz the radiation pattern is produced by the first patch, which is omnidirectional in the azimuth plane (xy) and has a balloon-like shape in the (elevation) E- and H-planes. The second patch does not contribute to the radiation at 38 GHz due to its small dimensions.

At 60 GHz, the radiation pattern is obtained by the second patch. On the other hand, the first patch contributes to the radiation through a higher-order mode, which results in a radiation pattern with undesirable nulls and side lobes. The cuts made in the first patch at the corners on the sides of the microstrip feed line are made to diminish the surface currents and slot fields near these regions and, thereby, prevent the formation of such higher-order modes in the cavity below the radiating patch. Thus, the radiation pattern at 60 GHz has a shape that is suitable for a diversity scheme that enhances the performance of a MIMO antenna system constructed from a number of properly allocated elements of such an antenna.

The geometry of the proposed dual-band microstrip patch antenna is illustrated in Figure 1, where $LP_{1},\;WP_{1}$ are the length and width of the first patch, respectively. Similarly, $LP_{2},\;WP_{2}$ are the length and width of the second patch, respectively, added to the length of the inset feed ($L_{ins}$), the gap width of the inset feed ($W_{ins}$), and the depth of the gap (GP) cut in the first patch. The dimensions are stated in Table 1. The proposed microstrip patch antenna is printed on Rogers $\mathrm{RO}3003^{\mathrm{TM}\;}$substrate of dielectric constant $\varepsilon_{r}=3.0$; dimensions of the substrate are 15 × 25 mm, height h=0.25 mm, and loss tangent δ=0.001. The conducting surface is made of copper with conductivity $\sigma =5.6\times 10^{7}\;S/m$.

## 3. Simulations and Results

In this section, the performance of the dual-band patch antenna is investigated and the corresponding numerical results are presented and discussed. The presented results are concerned with investigating the return loss and radiation patterns of the single element antenna using the commercially available CST Studio Suite^®^ software package.

### 3.1. Development of the Dual-Band Microstrip Patch antenna Design

**Figure 2:**
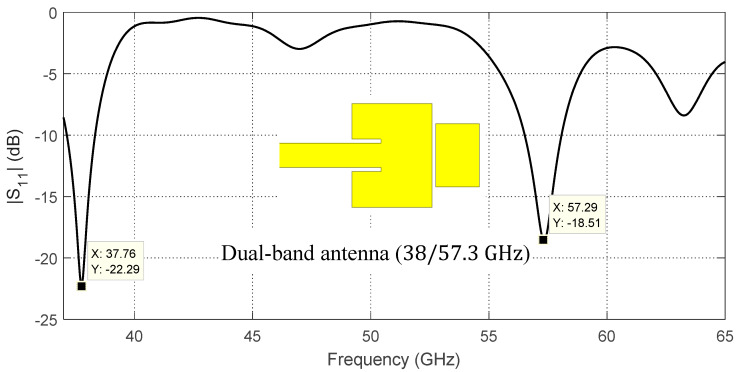
Simulated dependence of the return loss, $\left|S_{11}\right|$, on the frequency over a wide band of the frequency for the indicated dual-patch antenna (initial design).

It may be interesting to demonstrate the stages of the proposed antenna design to combine multiple radiation mechanisms to construct a single antenna operating in the dual-frequency band. Preliminarily, the simple idea of producing dual-band radiation can be implemented using two rectangular patches as shown in Figure 2.

**Figure 3:**
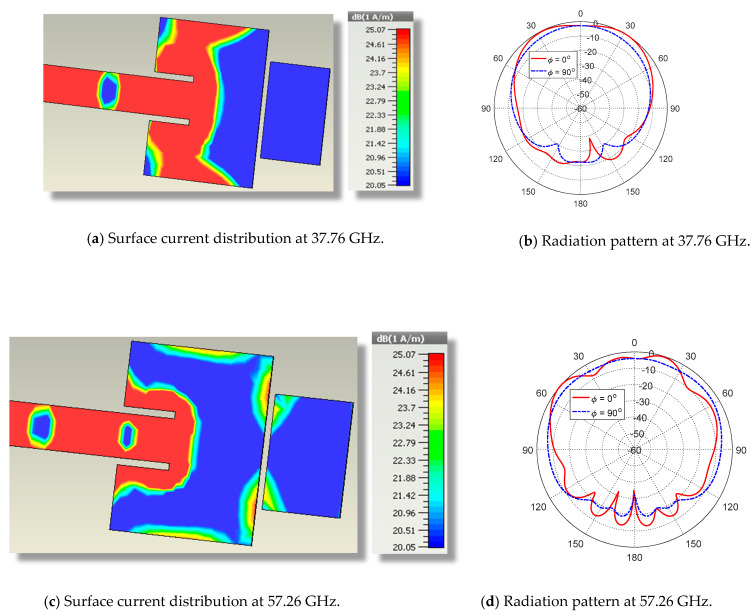
Simulated surface current distributions and the corresponding radiation patterns of the proposed dual-band patch antenna at 37.76 GHz and 57.26 GHz.

The mutual coupling between the two patches results in the frequency response of the return loss $\left|S_{11}\right|$ presented in Figure 2. The resonance frequencies of the two patches are shifted (particularly the resonance frequency of the upper band) due to the capacitive load caused by the other patch resulting in resonance frequencies of about 37.76 GHz and 57.26 GHz for the first and second patches, respectively. For further explanation of the radiation mechanisms at each frequency, the surface current distributions are presented in Figure 3a,c at 37.76 GHz and 57.26 GHz, respectively. The corresponding radiation patterns in the orthogonal elevation planes (ϕ=0° and ϕ=90°) are presented in Figure 3b,d. These radiation patterns have similar balloon-like shapes, which means that the radiation is omnidirectional in the azimuth planes.

Both resonance frequencies of the first patch (at 37.76 GHz and 57.26 GHz) are first-order, as the active region of the patch (the area over which the surface current has significant magnitude) at each of these frequencies is concentrated in the close vicinity of the junction between the feed line and the patch. Moreover, the area of the active region at each frequency is proportional to the square of the effective wavelength ($\lambda_{0}/\sqrt{\varepsilon_{r\;eff}}$). As a consequence, the radiation pattern at each of these frequencies has the conventional balloon-like shape of a rectangular patch radiating in its first-order resonance.

**Figure 4:**
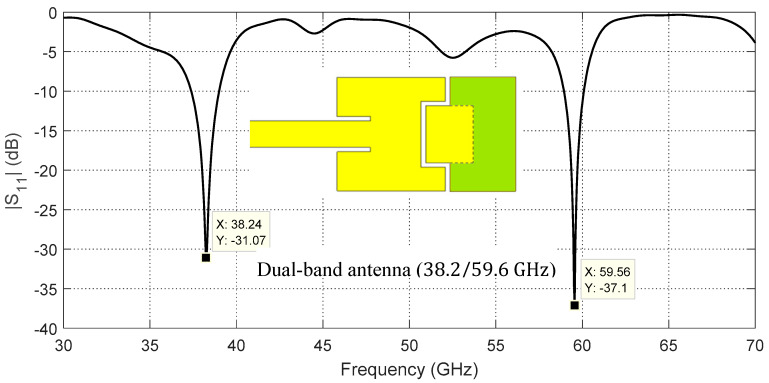
Simulated dependence of the return loss, $\left|S_{11}\right|$, on the frequency over a wide band of the frequency for the indicated dual-patch antenna (intermediate stage for the final design).

This design is modified by introducing an inductance to reduce the capacitive load caused by edge coupling between the two patches. This is achieved by subtending the second patch between two arms which are made as side extensions of the first patch as shown in Figure 4. The resulting “C” shape of the first patch can have the effect of a semi-turn producing the required compensating inductance which, in turn, reduces the reactive impedance caused by the capacitive edge coupling. For more inductive effect, the conducting part indicated as the green colored surface shown in Figure 4 is adhered to the second patch to complete the semi-turn made by the first patch and its extended arms. This modification results in the frequency response of the return loss, $\left|S_{11}\right|$, depicted in Figure 4. It is apparent that the resonance frequency at the center of the upper band is significantly corrected to be 59.56 GHz.

**Figure 5:**
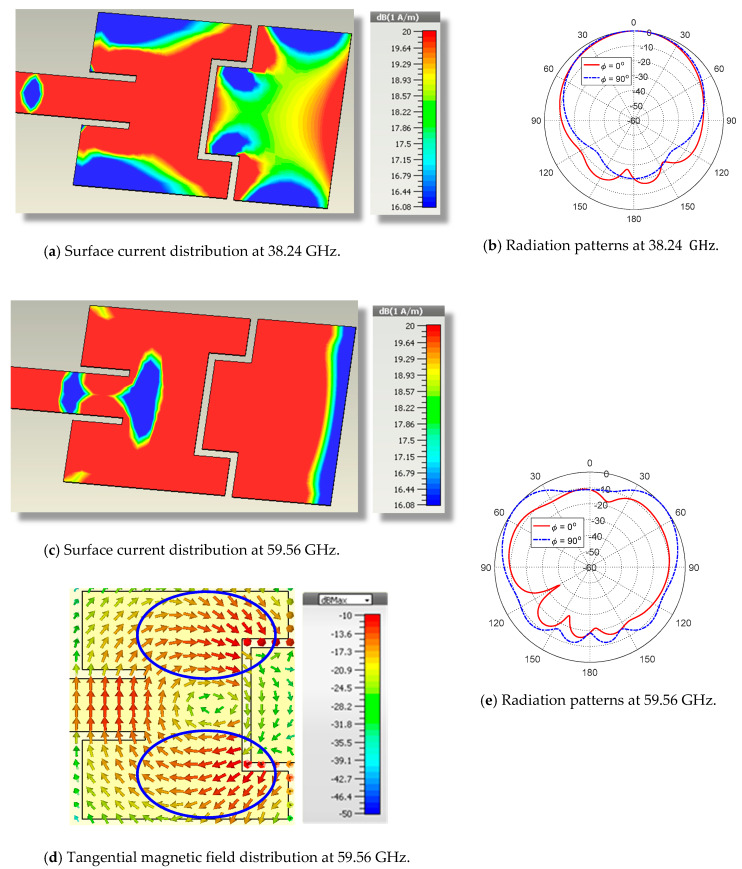
Simulated surface current, tangential magnetic field distributions, and the corresponding radiation patterns of the proposed dual-band patch antenna at 38.24 GHz and 59.56 GHz.

At  38.24 GHz, the dual-band antenna presented in Figure 4 has the surface current distribution presented in Figure 5a and the corresponding radiation patterns presented in Figure 5b. As shown in Figure 5b, the radiation pattern at 38.24 GHz has an acceptable balloon-like shape. This is attributed to the current distribution, which is still concentrated in a continuous region around the inset feed as shown in Figure 5a. On the other hand, the surface current, the tangential magnetic field distribution at 59.56 GHz, and the corresponding radiation patterns are presented in Figure 5c–e, respectively. Due to increasing the coupling between the first and second patches, the current flowing on the second patch is considerably increased in comparison to that presented in Figure 3c at the upper-frequency band. However, the radiation pattern of this patch at 59.56 GHz, shown in Figure 5e, is not acceptable. The difference between the maximum and minimum radiation in the elevation plane ϕ=90° is greater than 11 dB. In addition, the level of radiation in the plane ϕ=90° is globally much greater than the corresponding level in the plane ϕ=0°. This indicates that the radiation pattern is not omnidirectional in the azimuth planes, which can be made clear by mentioning that the surface current, which is distributed over a wide area, extends on both the first and second patches. Moreover, Figure 5d shows that the tangential magnetic field is mainly concentrated in two regions (surrounded by blue dashed oval shapes) where the tangential field has opposite horizontal directions leading to a considerable reduction of the radiated field in the direction normal to the patch surface (θ=0°). Hence, the antenna design presented in Figure 4 needs some modifications to increase the concentration of the surface current on the second patch (responsible for radiation in the upper-frequency band).

**Figure 6:**
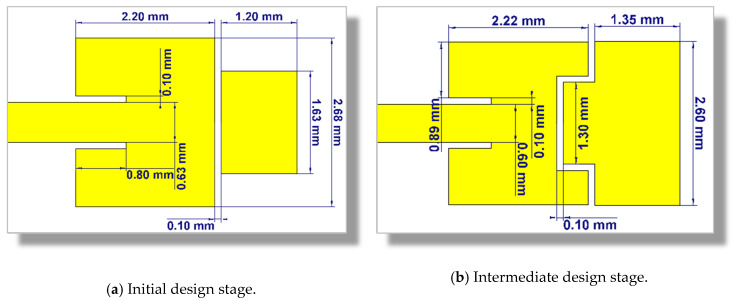
Dimensions of the dual-patch for the initial and intermediate design stages.

The dimensions of the dual-band patch antennas introduced in the initial and intermediate design stages are shown in Figure 6.

**Figure 7:**
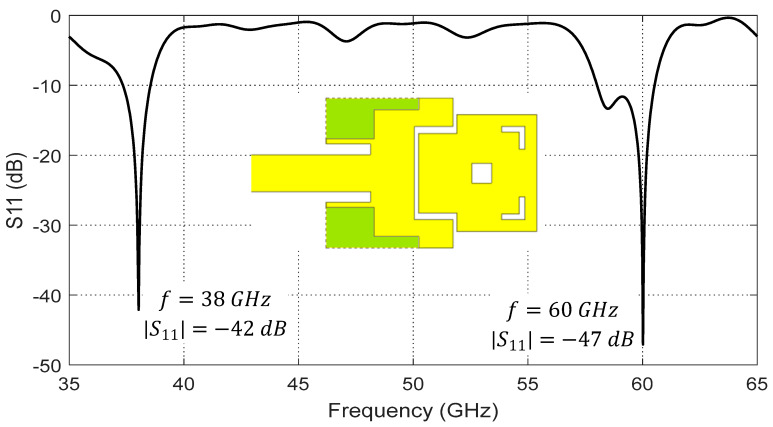
Simulated dependence of the return loss, $\left|S_{11}\right|$, on the frequency over a wide band of the frequency for the proposed dual-patch antenna (final design).

Further improvements to the geometry of the dual-band microstrip patch antenna can be made to achieve a radiation pattern shape at 60 GHz that is suitable for MIMO applications. As shown in Figure 7, some cuts in the first patch can be made at the corners on the sides of the microstrip feed line (shown in green color) to weaken the surface currents and slot fields near these regions. This prevents the formation of higher-order modes in the cavity below the radiating patch. Additionally, undesired grating lobes or nulls in the radiation pattern at 60 GHz resulting from possibly flowing currents on the large surface area of the second patch can be canceled by making other cuts in this patch. A central square gap and two corner-shaped slots can be cut as shown in Figure 7 to reduce the area of the active region of this patch by disturbing the paths of possible higher-order currents flowing near the corners. The geometrical parameters of the dual-patch antenna according to the final design are presented in Section 3.3 (after demonstrating a procedural parametric scan for selecting their optimal values).

These modifications of the antenna geometry result in the frequency response of the return loss, $\left|S_{11}\right|$, presented in Figure 7. As shown in this figure, the antenna impedance is perfectly matched at the two frequencies 38 and 60 GHz with return loss −42 dB and −47 dB, respectively. At 38 GHz, the bandwidth is about 1.2 GHz (37.34–38.54 GHz ), while, at 60 GHz, the bandwidth is about 2.52 GHz and can operate with matched impedance over the frequency range (58.01–60.53 GHz). The wide bandwidth at 60 GHz can be explained due to the second patch (responsible for radiation at this frequency) being excited through indirect feeding, where both capacitive and inductive coupling mechanisms are implemented. This leads to stabilizing the patch impedance over a much wider frequency band than that of the first patch, which is directly fed through a conventional microstrip line.

**Figure 8:**
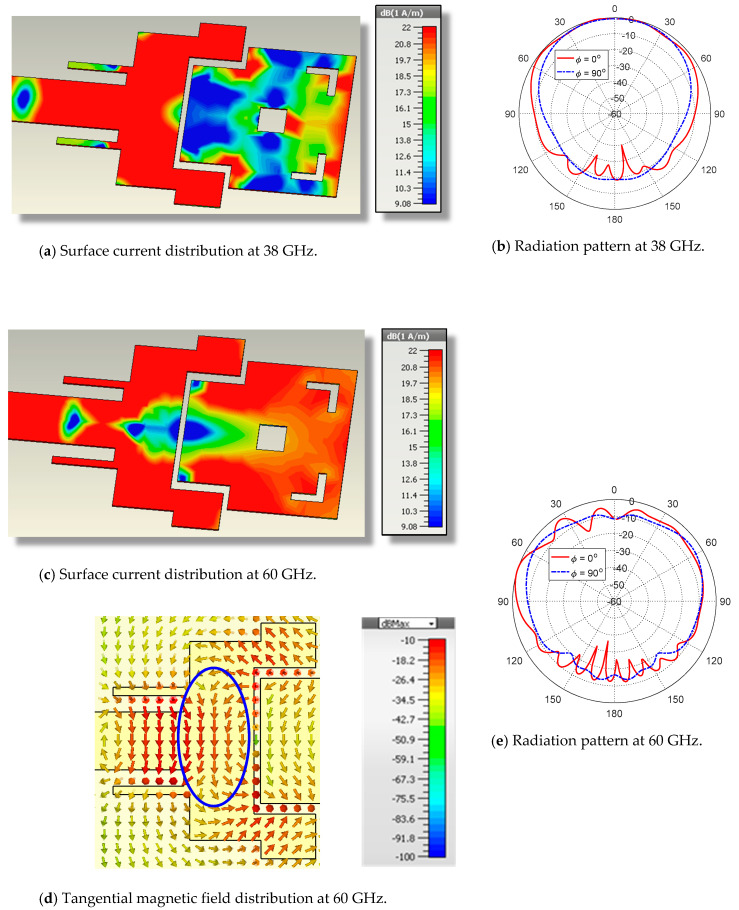
Simulated surface current, tangential magnetic field distributions, and the corresponding radiation patterns of the proposed dual-band patch antenna at 38 GHz and 60 GHz.

The surface current distributions and the corresponding radiation patterns at 38 and 60 GHz are presented in Figure 8. The radiation patterns for the proposed dual-band antenna at 38 GHz and 60 GHz are presented in Figure 8b,e, respectively, in the E-plane (ϕ=0°) and H-plane (ϕ=90°). It is obvious that the radiation patterns are almost balloon-like in the elevation planes and exhibit omnidirectional radiation in the azimuth planes. It should be noted that the geometrical modifications of the patch design shown in Figure 4 result in the tangential magnetic field being mainly concentrated in the central region (surrounded by blue dashed oval shape), as shown in Figure 8d where the magnetic field has unified vertical directions. When compared with the tangential magnetic field directions presented in Figure 5d, it becomes clear that the geometrical modifications of the patch have led to an increase in the radiated field in the direction normal to the patch surface (θ=0°), resulting in a more appropriate shape of the radiation pattern as shown in Figure 8e. The maximum gain is about 6.5 dBi at 38 GHz and 5.5 dBi at 60 GHz. Such radiation patterns (in both the lower- and upper-frequency bands) have suitable shapes for efficient diversity schemes when utilized to construct a 5G mobile handset MIMO system of properly allocated elements of such an antenna.

### 3.2. Parametric Study for the Selection of the Optimal Parameters of the Dual-Band Patch Antenna

The proposed dual-band microstrip patch antenna should satisfy the required operational and performance measures to be suitable as a 5G mobile handset antenna. The proposed patch antenna operates at 38 and 60 GHz with satisfactory performance, including impedance matching (low return loss), wide bandwidth, and proper shape of the radiation patterns in both the lower and upper bands of operation. This may be achieved using parametric study, including of the most important dimensional parameters of the patch antenna shown in Figure 1, such as the length and width of each radiating patch ($LP_{1},\;WP_{1}$ for the first patch, $LP_{2},\;WP_{2}$ for the second patch), length of the inset feed ($L_{ins}$), gap width for the inset feed ($W_{ins}$), and the depth of the gap (GP) cut in the first patch.

#### 3.2.1. Effect of the Parasitic Patch Width

**Figure 9:**
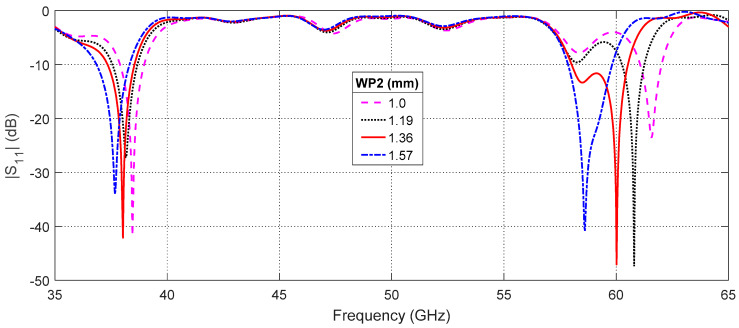
Changing the width of the second patch$,\;WP_{2}$, leads to coarse tuning of the center frequency of the upper band (60 GHz) and fine tuning of the center frequency of the lower band.

The center frequency of the upper band (around 60 GHz) of the proposed patch antenna is very sensitive to any change in the second patch width,$\;WP_{2}$. This is clear in Figure 9, which presents the frequency response of the return loss, $\left|S_{11}\right|$, for different values of $WP_{2}$. The center frequency of the upper band changes from $f_{U}=58.6\;\mathrm{GHz}$ for $WP_{2}=1.0\;\mathrm{mm}$ to $f_{U}=61.6\;\mathrm{GHz}$ for $WP_{2}=1.57\;\mathrm{mm}$. Moreover, the center frequency of the lower band $f_{L}$ has a weak dependence on $WP_{2}$ such that it changes from $f_{L}=37.7\;\mathrm{GHz}$ for $WP_{2}=1.0\;\mathrm{mm}$ to $f_{L}=38.5\;\mathrm{GHz}$ for $WP_{2}=1.57\;\mathrm{mm}$. Thus, changing this parameter enables the tuning of the center frequencies of the lower and upper bands. As shown in Figure 9, setting $WP_{2}=1.36\;\mathrm{mm}$ results in satisfying the required frequencies $f_{L}=38\;\mathrm{GHz}$ and $f_{U}=60\;\mathrm{GHz}$ at the same time.

#### 3.2.2. Effect of the Inset Length

**Figure 10:**
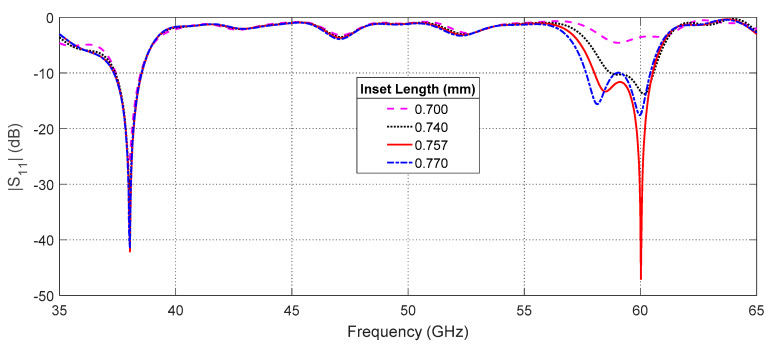
Effect of changing $L_{ins}$ on the frequency response of the return loss $\left|S_{11}\right|$ at the antenna input port relative to 50  Ω characteristic impedance of the microwave source.

One of the antenna design parameters that has a great effect on the input impedance of the patch antenna is the feeding inset length, $L_{ins}$; see Figure 1. The effect of changing $L_{ins}$ on the frequency response of the return loss, $\left|S_{11}\right|$, at the antenna input port relative to 50  Ω characteristic impedance of the microwave source is presented in Figure 10. As shown in this figure, $\left|S_{11}\right|$ is strongly dependent on $L_{ins}$ at 60 GHz for the indicated range of lengths. The optimum value of $\left|S_{11}\right|$ is −47 dB and is obtained for$\;L_{ins}=0.757\;\mathrm{mm}$. On the other hand, the value of $\left|S_{11}\right|$ at 38 GHz seems to be independent of $L_{ins}$ (through the indicated range of lengths).

### 3.3. Optimal Design of the Dual-Band Microstrip Patch Antenna

**Figure 11:**
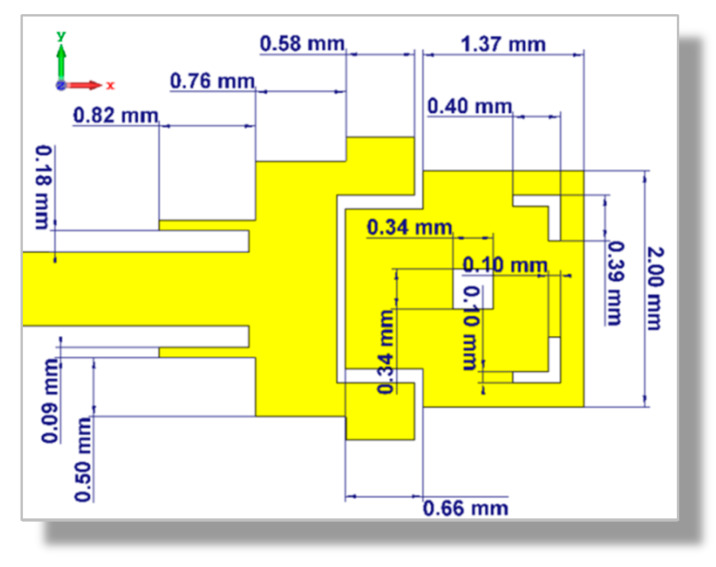
Optimum dimensional parameters of the dual-band microstrip patch antenna for operation.

According to the parametric study performed in Section 3.2, the dimensional design parameters to achieve the best performance of the proposed dual-band patch antenna are indicated in Figure 11. The following subsections are concerned with the theoretical and experimental investigations of the return loss and the radiation patterns of the dual-band antenna with its optimal design parameters.

**Figure 12:**
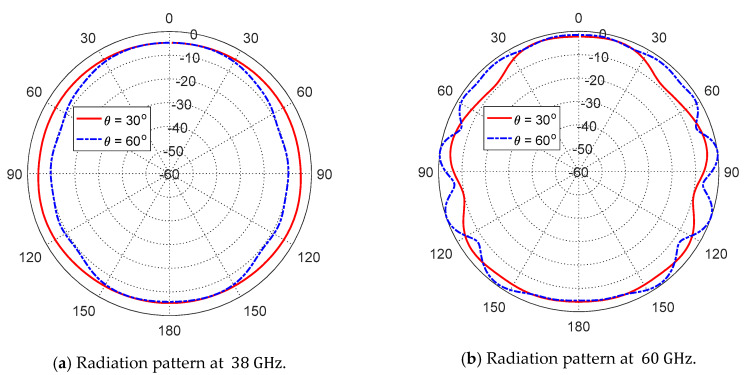
Simulated radiation patterns in the azimuth planes for the dual-band microstrip patch antenna whose dimensions are shown in Figure 11.

The azimuth radiation patterns for the proposed dual-band antenna at 38 GHz and 60 GHz are presented in Figure 12a,b, respectively, in the planes θ=30° and θ=60°. As shown in the figures, the radiation patterns are almost omnidirectional in the azimuth planes.

The proposed dual-band antenna with the optimized design has the radiation and total efficiencies listed in Table 2 at the center frequencies of the lower and upper bands of operation. The antenna efficiency at the lower frequency is better than that at the higher frequency as the losses in the dielectric substrate increase with the frequency.

### 3.4. Dual-Band Patch Antenna Fabrication and Experimental Assessment

This section is concerned with the presentation of the experimental measurements of the dual-band microstrip patch antenna. To confirm the accuracy of the assessed performance for the proposed antenna, the measurement results are compared to those obtained by electromagnetic simulation using the CST^TM^ software package.

#### 3.4.1. Fabrication of the Antenna Prototype

**Figure 13:**
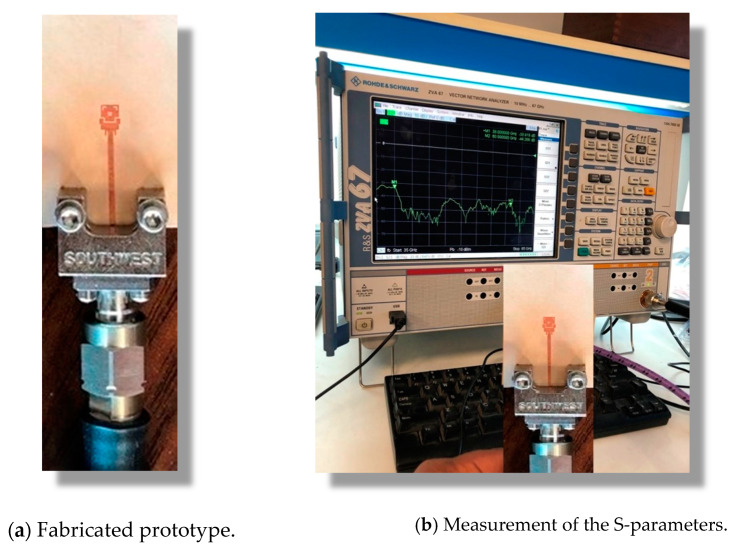
Measurement of the scattering parameter $\left|S_{11}\right|$ of the proposed dual-band patch antenna using the vector network analyzer (VNA; Rhode and Schwartz model ZVA67).

The prototype shown in Figure 13a was fabricated for the purpose of experimental assessment of the performance of the proposed dual-band microstrip patch antenna. The circuit was fabricated by photolithography. A photomask of the circuit layout was prepared and the top layer of the copper-coated substrate was covered with photoresist by spin coating. The photomask was placed next to the top layer of the substrate and exposed to an intense UV light to remove the photoresistant layer from the unwanted copper areas. In etching, a liquid chemical agent removed the uppermost layer of the substrate in the areas that were not protected by the photoresistant layer.

#### 3.4.2. Measurements of the Return Loss

A vector network analyzer (VNA; Rhode and Schwartz model ZVA67) was used for measuring the frequency response of the return loss $S_{11}$ and the corresponding voltage standing wave ratio (VSWR). A 1.85 mm end-launch connector from Southwest Microwave Inc. was used for connecting the antenna to the VNA as shown in Figure 13b.

**Figure 14:**
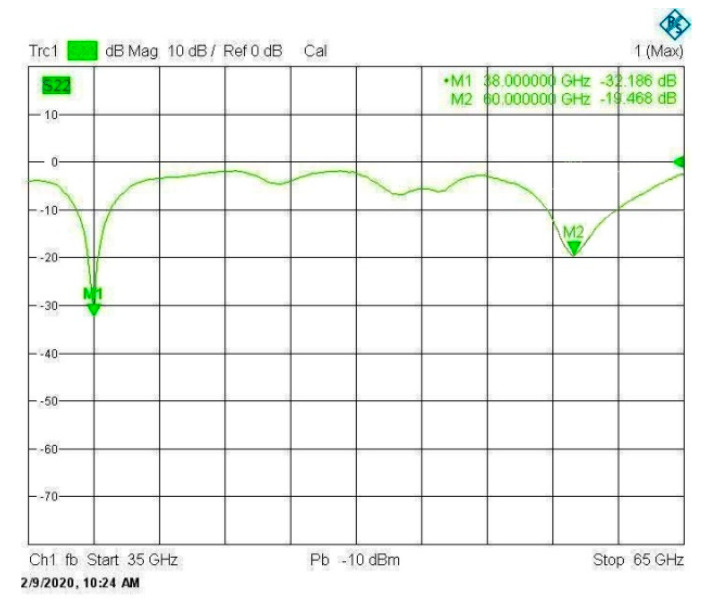
The measured frequency response of the scattering parameter $\left|S_{11}\right|$ of the VNA.

**Figure 15:**
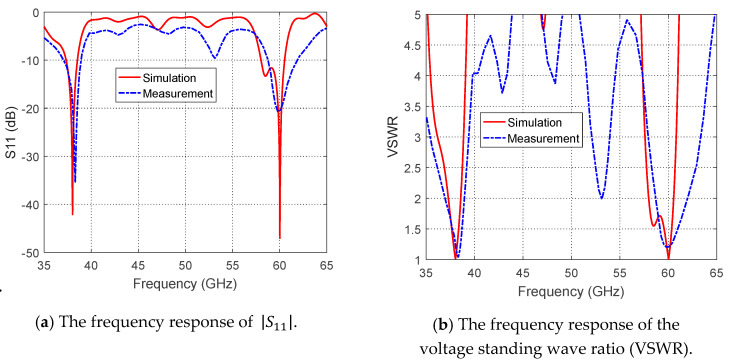
Measured frequency responses of the return loss, $\left|S_{11}\right|$, and the corresponding VSWR of the proposed dual-band microstrip patch antenna (relative to 50 Ω characteristic impedance of the microwave source) compared with the simulation results.

The frequency response of the return loss $\left|S_{11}\right|$ as measured by the VNA is presented in Figure 14. Comparisons between the simulation and measurement results for the frequency dependencies of $\left|S_{11}\right|$ and the corresponding VSWR are presented in Figure 15, and show excellent agreement. The impedance matching bandwidths (for $\left|S_{11}\right|<-10\mathrm{dB}$) obtained through measurements are shown to be better than those obtained by simulation. At 38 GHz, the measured bandwidth is about 2.0 GHz, whereas the simulated bandwidth is about 1.2 GHz. In addition, at 60 GHz, the measured bandwidth is about 3.2 GHz, whereas the simulated is about 2.52 GHz. Moreover, the upper-frequency bandwidth obtained by measurements is shown to be centered at about 60 GHz, whereas the upper band obtained by simulation is shown to be shifted and centered at about 59.5 GHz.

#### 3.4.3. Measurement of the Radiation Patterns and Maximum Gain

**Figure 16:**
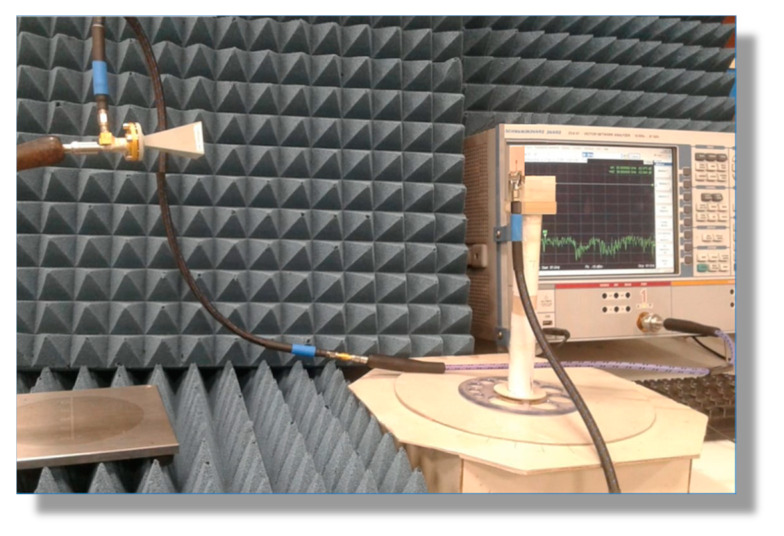
Experimental setup for measuring the radiation pattern and gain of the dual-band antenna.

The experimental setup for measuring the radiation patterns and the maximum gain of the proposed antenna is presented in Figure 16. The VNA operating in two-port measurement mode was used for this purpose by measuring the transmission coefficient$\;\left|S_{21}\right|$ through the antenna under test and the reference-gain linearly-polarized horn antennas, models LB-018400 (for 38 GHz band) and LB-12-10-A (for 60 GHz band).

**Figure 17:**
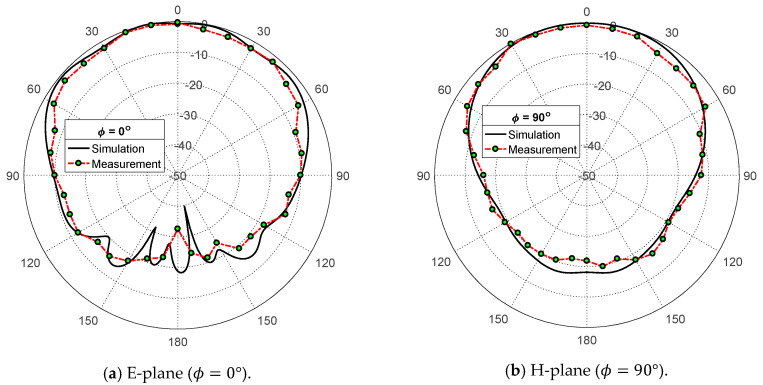
Measured radiation patterns of the proposed dual-band microstrip patch antenna at 38 GHz compared with the simulation results.

**Figure 18:**
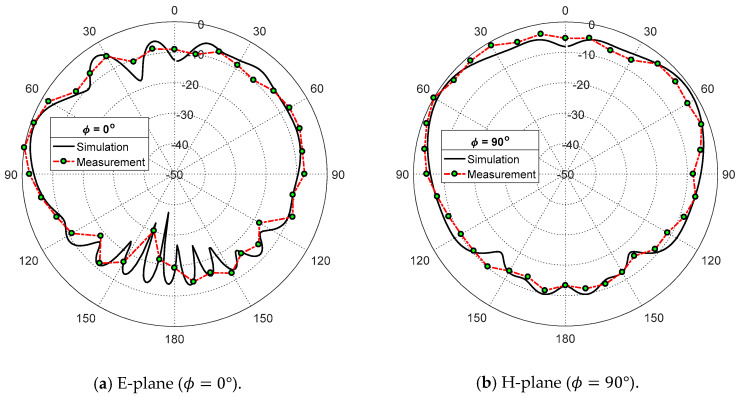
Measured radiation patterns of the proposed dual-band microstrip patch antenna at 60 GHz compared with the simulation results.

The measured radiation patterns in the elevation planes ϕ=0° and ϕ=90° corresponding to the E-plane and H-plane, respectively, of the proposed patch antenna, are presented in Figure 17 and Figure 18 at the frequencies 38 GHz and 60 GHz, respectively. It is shown that the measured and simulated radiation patterns are close to each other and show good agreement. The measured maximum gain values are 6.2 dBi and 4.9 dBi at 38 GHz and 60 GHz, respectively, which are close to the measured values (6.5 dBi and 5.5 dBi at 38 GHz and 60 GHz, respectively).

### 3.5. Performance Comparison with Published Work

**Figure 19:**
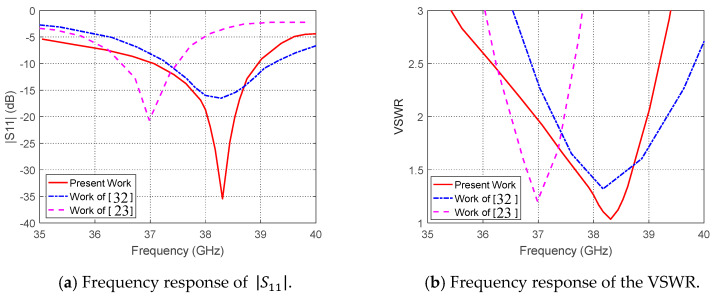
Comparisons between the frequency responses of the $\left|S_{11}\right|$ and VSWR obtained in the present work for the proposed dual-band antenna and those obtained in [23] and [32] at 38 GHz.

The performance of the proposed dual-band microstrip regarding the impedance matching and the corresponding bandwidth can be compared to other published results for comparative assessment. For example, the frequency response of the measured $\left|S_{11}\right|$ for the antenna proposed in the present work is compared to that obtained in [23] and [32] as shown in Figure 19a. The corresponding frequency responses of the VSWR are compared to each other as shown in Figure 19b. The minimum value of the return loss achieved in the present work is about −35 dB (VSWR≈1) whereas that achieved in [32] is about −17 dB (VSWR≈1.3) and that achieved in [23] is about −21 dB (VSWR≈1.2). Moreover, for the antenna proposed in the present work, the bandwidth (BW) of operation for $\left|S_{11}\right|<-10\;\mathrm{dB}$ is about 2 GHz and is centered exactly at 38 GHz, whereas that obtained in [32] is centered at about 38.3 GHz (a shift of about 300 MHz) with nearly the same bandwidth. The impedance matching bandwidth obtained in [23] is about 1 GHz and centered at 37 GHz (with about 1 GHz frequency shift from the desired frequency).

**Figure 20:**
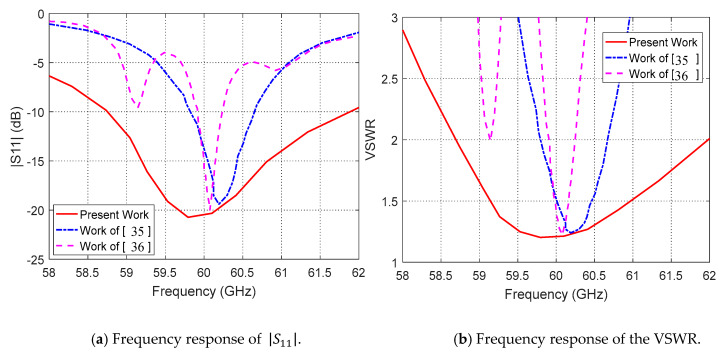
Comparisons between the frequency response of the $\left|S_{11}\right|$ obtained in the present work for the proposed dual-band antenna and those obtained in [35] and [36] at 60 GHz.

In the upper band centered at 60 GHz, the frequency response of $\left|S_{11}\right|$ measured for the antenna proposed in the present work is compared to those presented in [35] and [36] as shown in Figure 20. The minimum value of the return loss achieved in the present work is about −21 dB, whereas those achieved in [35] and [36] are about −19 dB and −20 dB, respectively. The impedance matching bandwidth achieved in the present work is about 3.3 GHz (for VSWR≤2.0 or, equivalently, $\left|S_{11}\right|\leq -10\;\mathrm{dB}$), whereas those achieved in [35] and [36] are 0.9 GHz and 0.35 GHz, respectively.

A comparative summary with the antenna performance achieved in [23,32,35,36] is listed in Table 3. It is shown that the dual band microstrip patch antenna proposed in the present work has superior performance regarding the impedance matching bandwidth in all the cases, and particularly for stringent matching conditions (VSWR≤1.5 and VSWR≤1.25).

## 4. Conclusions

The present work introduces a microstrip patch antenna with a novel design to operate in the 38/60 GHz dual-band. The single antenna is constructed as first and second rectangular patches with some geometrical modifications to achieve perfect impedance matching and balloon-like radiation patterns over the lower- and upper-frequency bands of operation. A microstrip line is the main feeder for the first patch, which is responsible for the lower band (38 GHz) radiation, and capacitive and inductive feeding are responsible for the upper band (60GHz) radiation. The performance of the single-element antenna is assessed through numerical and experimental investigations. It is shown that the simulation results agree with the experimental measurements and both show the good performance of the proposed dual-band patch antenna. The bandwidths achieved around 38 GHz and 60 GHz are about 2.0 GHz and 3.2 GHz, respectively. The return loss has minimum values of −42 dB and −47 dB at 38 GHz and 60 GHz, respectively. The maximum gain is 6.5 dBi and 5.5 dBi at 38 GHz and 60 GHz, respectively. The experimentally assessed frequency band of the proposed antenna is shown to be better than that obtained by other published work for operation at 38 GHz.

## Figures and Tables

**Table 1:** Dimensions of the dual-patch microstrip patch antenna.

Dimension	$LP_{1}$	$LP_{2}$	$WP_{1}$	$WP_{2}$	GP	$L_{ins}$	$W_{ins}$
**Value (mm)**	2.165	1.22	2.57	1.36	0.66	0.76	0.18

**Table 2:** Radiation and total efficiencies of the dual-band microstrip patch antenna whose dimensions are shown in Figure 11 (The listed values have been obtained through electromagnetic simulation using CST^®^).

	Frequency	
	38 GHz	60 GHz
Total Efficiency	89.53%	79.42%
Radiation Efficiency	89.57%	79.87%

**Table 3:** Comparison between the bandwidth achieved in the present work to that achieved in other published work.

Performance Measure		Performance at 38 GHz			Performance at 60 GHz		
		Present Work	Work of [32]	Work of [23]	Present Work	Work of [35]	Work of [36]
**Center Frequency**		38.0	38.3	37.0	60.5	60.3	60.1
**BW**	VSWR≤1.25	2.0	2.0	1.0	3.3	0.9	0.35
	VSWR≤ 2.0	1.0	0.75	0.45	1.85	0.5	0.2
	VSWR≤1.5	0.5	0	0.07	0.8	0	0.02
**Patch area (mm^2^)**		2.0×3.0	1.0×1.0	3.55×3.55	2.0×3.0	1.4×1.4	Array only
**Patch Gain (dBi)**		6.5	7.4	7.7	5.5	6.2	NA
